# Advances and challenges in cirrhosis and portal hypertension

**DOI:** 10.1186/s12916-017-0966-6

**Published:** 2017-11-10

**Authors:** Annalisa Berzigotti

**Affiliations:** Swiss Liver Center, Hepatology, University Clinic for Visceral Surgery and Medicine (UVCM), Inselspital, University of Bern, MEM F807, Murtenstrasse 35, CH, 3010 Berne, Switzerland

**Keywords:** Hepatic venous pressure gradient, Non-invasive methods, Liver stiffness, Therapy, Transjugular intrahepatic portosystemic shunt

## Abstract

**Background:**

Liver cirrhosis is the fourth cause of death in adults in Western countries, with complications of portal hypertension being responsible for most casualties. In order to reduce mortality, development of accurate diagnostic methods for early diagnosis, effective etiologic treatment, improved pharmacological therapy for portal hypertension, and effective therapies for end-stage liver failure are required.

**Discussion:**

Early detection of cirrhosis and portal hypertension is now possible using simple non-invasive methods, leading to the advancement of individualized risk stratification in clinical practice. Despite previous assumptions, cirrhosis can regress if its etiologic cause is effectively removed. Nevertheless, while this is now possible for cirrhosis caused by chronic hepatitis C, the incidence of cirrhosis due to non-alcoholic steatohepatitis has increased dramatically and effective therapies are not yet available. New drugs acting on the dynamic component of hepatic vascular resistance are being studied and will likely improve the future management of portal hypertension.

**Conclusion:**

Cirrhosis is now seen as a dynamic disease able to progress and regress between the compensated and decompensated stages. This opinion article aims to provide the author’s personal view of the current major advances and challenges in this field.

## Background

Chronic liver disease (CLD) affects more than 29 million people in Europe [1] and over 300 million people worldwide. The main causes of CLD are alcohol abuse, chronic viral hepatitis, and metabolic factors (non-alcoholic fatty liver disease). Over time, extracellular fibrotic tissue develops and accumulates in the liver as a result of chronic injury, progressively leading to fibrous septa that prevent normal oxygenation and blood exchange to the liver parenchyma. This late stage, featuring marked liver anatomical changes, including hepatocyte extinction, micro- and macrovascular remodeling, neoangiogenesis, nodule formation, and development of portosystemic shunts, is termed ‘cirrhosis’ [2]. Mortality in CLD is primarily due to complications of liver cirrhosis and hepatocellular carcinoma (HCC), which is considerably more prevalent in patients with cirrhosis. The term ‘advanced chronic liver disease’ (ACLD) has been recently proposed to better mirror the late stages of CLD, which should be considered within a continuum spectrum, ranging from severe fibrosis to fully developed cirrhosis [3].

## Compensated versus decompensated cirrhosis: the burden of advanced chronic liver disease (ACLD)

According to the largest study available thus far [4], cirrhosis represents the fourth cause of death due to non-communicable diseases worldwide, with the total number of deaths from cirrhosis and liver cancer having steadily risen by approximately 50 million per year over the last two decades. This large mortality rate is due, to some extent, to a late diagnosis. A decades-long asymptomatic stage during which no overt sign of the disease is noticed is characteristic of CLD. Indeed, even after the onset of cirrhosis, the disease can remain asymptomatic, or ‘compensated’, for a long time [5]. Nevertheless, during this time, portal hypertension progressively develops, usually accompanied by a decline in hepatocellular function.

**Fig. 1:**
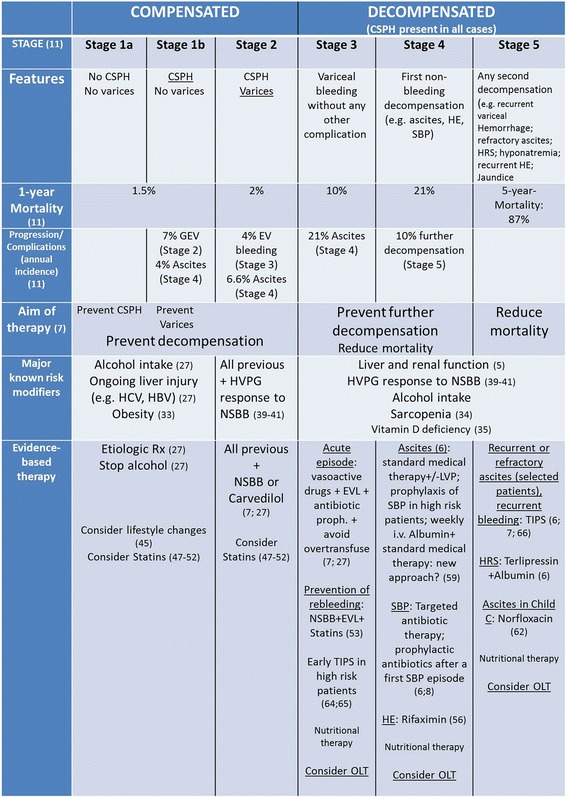
Clinical stages of cirrhosis. The first major classification is based on the absence or presence of complications. Cirrhosis is named ‘compensated’ in the absence of complications, and ‘decompensated’ if complications are present or have been present in the past. In patients with compensated cirrhosis, the presence of clinically significant portal hypertension (HVPG ≥ 10 mmHg) identifies a substage with higher risk of developing any complication (varices, decompensation). The decompensated stage is characterized by a high risk of progression to further decompensation, liver failure, and death. Evidence-based therapy has been developed by targeting the pathophysiological mechanisms driving the transition from a given step to the following one. The major advances in each stage are indicated within the figure

Portal hypertension is the major driver in the transition from the compensated to the ‘decompensated’ stage of cirrhosis [5], defined by the presence of clinical complications, including ascites [6], bleeding from gastroesophageal varices [7], spontaneous bacterial peritonitis [8], hepatorenal syndrome [6], and hepatic encephalopathy [9]. Further decompensating episodes are often triggered by bacterial infections [10], and are associated with a very high mortality risk. From a prognostic point of view, compensated and decompensated cirrhosis are dramatically different, and can be considered as two separate diseases. Furthermore, within these two major stages, several sub-stages with varying risk of further decompensation and death can be identified [11] (Fig. 1). Knowledge of the pathophysiological mechanisms driving the transition within these stages is key in the current management of cirrhosis [7]. Besides its negative impact on life expectancy, cirrhosis implies several other burdens, including a marked increase in healthcare costs due to hospitalization and treatment (estimated at approximately $2.5 billion per year in the US) [12], loss of productivity (estimated at $10.6 billion per year in the US) [12], and a marked reduction in quality of life [13]. These burdens are almost exclusively caused by complications during the decompensated stage.

Given that chronic viral hepatitis C (HCV) is a leading etiology of CLD [14], the recent availability of direct, high efficacy oral antiviral agents against HCV represents a major breakthrough towards achieving a reduction in mortality linked to CLD. Unfortunately, despite the reduction in the incidence of HCV-related liver disease complications already observed [15] and the marked decrease further expected over the coming years, over 40% of HCV infection cases have not been identified and may be recognized at a late, decompensated stage, when treatment of the viral infection may be futile [16]. In addition, other etiologies of CLD are becoming more common or remaining steadily frequent. Cirrhosis due to non-alcoholic steatohepatitis is markedly increasing as a consequence of the obesity pandemic worldwide [17], already ranking second among the etiologies of cirrhosis in patients on the waiting list for liver transplantation in the US [15]. Furthermore, liver disease associated with alcohol use disorders is highly prevalent worldwide, and is particularly relevant in Europe, where it accounts for the highest proportion of cirrhosis cases [18]. Of note, over the past 30 years, mortality due to cirrhosis in Europe increased in areas with the highest alcohol consumption (United Kingdom, Eastern Europe, Ireland, and Finland) [19]. Nevertheless, in several countries within the EU limiting alcohol use is not yet considered an absolute priority for policy-makers [20].

Over the last decades, new knowledge on the pathophysiology, diagnostic methods, and therapy of cirrhosis and portal hypertension have significantly improved the management of this disease, with a marked reduction in mortality related to some of its complications, particularly variceal bleeding [21]. However, in a recent analysis based on over 100,000 cases, 30-day mortality following discharge for any decompensation of cirrhosis was equal to or even higher than that observed 10 years prior, suggesting that the burden of mortality was merely shifted to the immediate postdischarge period [22]. Among the major determinants of mortality are inflammation in acute-on-chronic liver failure (associated with different complications of end-stage liver disease) and HCC [23] (not discussed in the present paper), both of which have been the subject of extensive research but remain unsatisfactorily resolved.

**Fig. 2:**
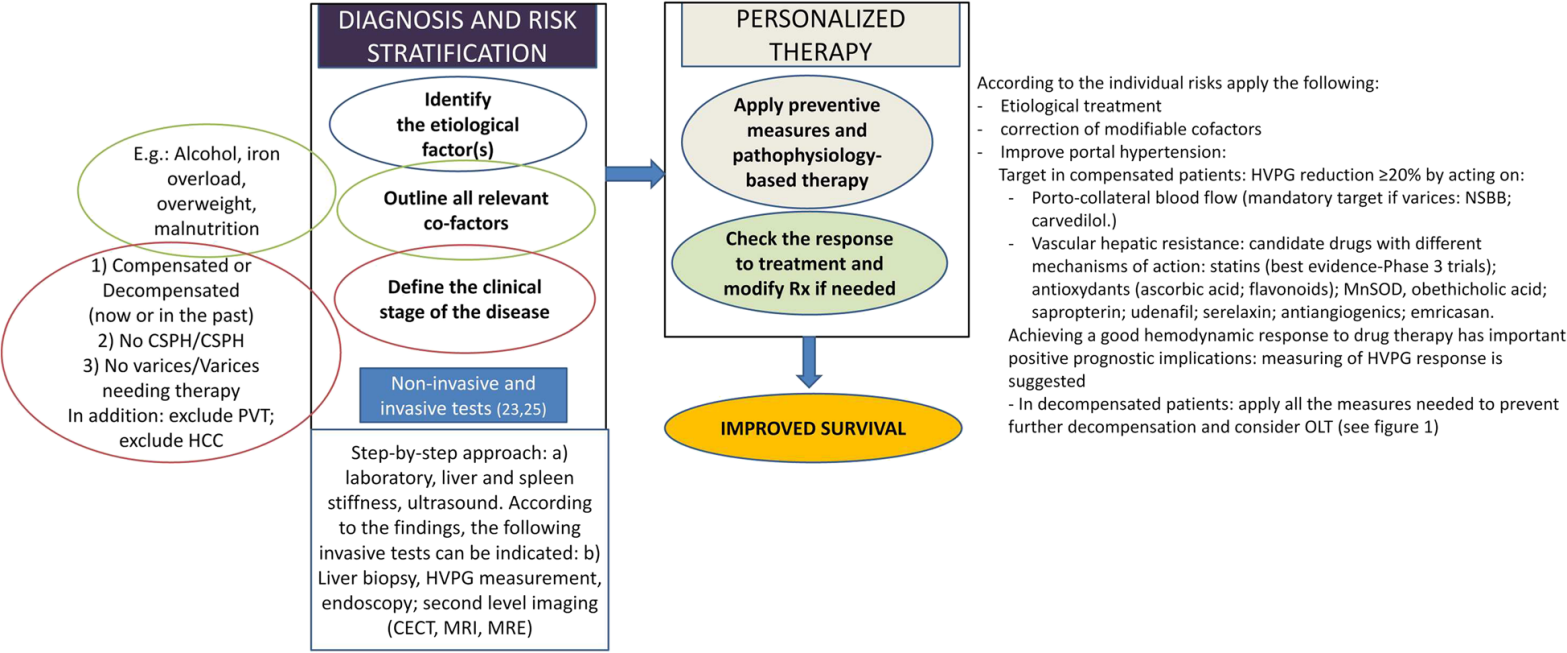
Logical steps in the clinical management of advanced chronic liver disease/cirrhosis. Improved survival can be achieved through adequate diagnosis and risk stratification, thus allowing a personalized approach to therapy. Some examples of factors to be considered, as well as the major pathophysiological factors driving the therapy of portal hypertension in patients with compensated cirrhosis, are provided

To achieve a substantial improvement in survival, every step of the management process of patients with ACLD should be addressed and optimized (Fig. 2). An early diagnosis of cirrhosis, i.e., within the compensated stage, and an accurate risk stratification are key to the following steps. Indeed, in the author’s opinion, the use of resources at this initial step (e.g., initiation of HCC surveillance, endoscopic screening of varices needing treatment in patients at high risk, prevention of decompensation by appropriate non-pharmacological and pharmacological therapy) is largely justified by the expected survival benefits.

## Early diagnosis and risk stratification: moving towards personalized medicine

The reference standard methods to diagnose cirrhosis, portal hypertension, and esophageal varices are liver biopsy, hepatic venous pressure gradient (HVPG) measurement, and endoscopy, respectively [24]. All of these methods are invasive, and require expertise to be correctly performed and interpreted. Undoubtedly, the availability of novel non-invasive diagnostic methods, and ultrasound elastography in particular, has enhanced the likelihood of early diagnosis of ACLD, facilitating the identification of patients with compensated disease who are at high risk of complications, prior to the occurrence of decompensation. Liver stiffness (and more recently spleen stiffness) can be measured by various ultrasound elastography methods [25], and mirrors the severity of liver disease and portal hypertension in patients with compensated ACLD [26]. The diagnosis of clinically significant portal hypertension (CSPH; HVPG ≥ 10 mmHg) is made possible by elastography, with an accuracy greater than 80% when using a binary cut-off approach [27]. As with all numerical variables holding prognostic value, liver stiffness can be modeled and calibrated, and the risk (probability) of CSPH can be calculated according to the measured values [28], thus leading to personalized medical decision-making. The vast data available regarding the relationship between liver stiffness, CSPH, and varices led the Baveno VI consensus conference on portal hypertension, held in 2015 [29], to suggest a simple combination of liver stiffness measured by transient elastography (<20 kPa) and platelet count (>150 G/L) in order to identify patients at low risk of varices needing treatment in whom endoscopic screening could be safely avoided [29]. Since 2015, these non-invasive criteria have proven robust and accurate, even if conservative (only approximately 20–25% of endoscopies spared). Recent research has proposed expanded non-invasive criteria allowing a much larger proportion of endoscopies to be spared without increasing the risk of false negative results [30, 31].

Real-time, simple diagnostic methods, such as ultrasound and elastography, are key to achieving bedside screening and first risk stratification. However, in patients who cannot be sufficiently characterized by these simple methods or in particularly sensitive situations, such as in patients with compensated cirrhosis and potentially resectable HCC [23, 32], HVPG measurement remains the best method to accurately stage portal hypertension. Nevertheless, recent advances in magnetic resonance imaging (magnetic resonance elastography [33], multiparametric magnetic resonance imaging [34]) hold promise and should be further investigated as surrogates of portal hypertension, particularly in patients who are not appropriate candidates for ultrasound elastography.

Despite the use of diagnostic tests being of paramount importance to achieve a correct risk stratification, the meaning of risk factors that are easily detected by physical examination and clinical history should not be disregarded. For instance, factors related to nutrition, and which are therefore potentially modifiable, should be actively investigated. Irrespective of the etiology leading to ACLD, overweight and obesity are increasingly observed in compensated patients [35], and have been consistently associated with an up to three-fold higher risk of clinical decompensation. Further, sarcopenia [36] and vitamin D deficiency [37] are frequent in cirrhosis (including in obese patients), almost invariably present in decompensated patients, and associated with higher mortality. Research in the field of nutritional factors modulating the natural history of cirrhosis is insufficient and represents a field for future investigation. For example, while alcohol intake is a well-known negative prognostic factor, coffee consumption has only recently been proven protective [38, 39].

Future research should also focus on providing accurate and individualized prediction of ‘hard’ endpoints, such as clinical decompensation and death, by non-invasive diagnostic methods. In the author’s opinion, the development of risk algorithms similar to those used in cardiovascular medicine (e.g., Framingham risk score [40]) would be advisable and feasible in the field of compensated cirrhosis to predict and stratify the risk of complications of portal hypertension.

## Advances in therapy

Several studies have demonstrated that, in portal hypertensive patients, if portal pressure is reduced enough (i.e., by at least 20%) by applying pharmacological and/or non-pharmacological therapies, the risk of decompensation or further decompensation and death is markedly reduced [7, 41–43] – this constitutes the rationale of treatment of portal hypertension. To achieve the highest efficacy, treatment should be aimed at correcting the main pathophysiological target in each stage of cirrhosis. In the early, compensated stages of cirrhosis, increased hepatic resistance plays a pivotal role in the development of portal hypertension (Pressure = Resistance × Flow) [2]. Therefore, in compensated cirrhosis, correction of increased intrahepatic resistance should be addressed [7, 44]. This can be achieved by ameliorating the mechanical component of resistance mostly represented by fibrosis and/or by acting on the functional component represented by active vasoconstriction and sinusoidal endothelial dysfunction [45]. Etiologic treatments have been shown effective in improving fibrosis and can lead to cirrhosis regression in the long term [46]; thus, they should be considered central at this stage of the disease.

Short-term (4 months) lifestyle changes consisting of diet and exercise combinations are able to improve obesity in compensated cirrhosis and are associated with a significant reduction in HVPG [47], likely mirroring a decrease in intrahepatic resistance (e.g., mediated by a decrease in insulin resistance). While supplementing vitamin D deficiency and correcting sarcopenia is likely to positively influence prognosis, the mechanisms driving the interaction between nutritional factors and portal hypertension remain to be elucidated.

Pure antifibrotic drugs are currently lacking [48]. However, statins, which improve the phenotype of sinusoidal endothelial cells by restoring nitric oxide production, are able to decrease intrahepatic fibrogenesis and angiogenesis in experimental models [49] and ameliorate portal hypertension by decreasing both the dynamic and structural components of intrahepatic resistance [50]. Interestingly, this is accompanied by an amelioration in hepatic function and perfusion in patients with cirrhosis [51]. Statins have proven effective in preventing hepatic decompensation in large epidemiological surveys in patients with HCV and hepatitis B virus cirrhosis [52, 53]. In addition, their use has been associated with a decreased risk of HCC [54] and, most recently, addition of simvastatin to standard medical and endoscopic therapy has been shown to improve survival in a double-blind randomized multicenter clinical trial in patients who survived an episode of bleeding from esophageal varices [55]. Thus, statins constitute the most promising class of drugs to be added to the standard therapy armamentarium for ACLD and portal hypertension.

Once CSPH has developed, and even more so following the formation of varices, the resulting hyperdynamic circulatory state leads to an increased portocollateral flow, which aggravates portal hypertension [2, 56]. At this stage, drugs acting to reduce blood flow are effective in reducing portal pressure. Non-selective beta-blockers (NSBBs; propranolol, nadolol, or carvedilol) are the mainstay of therapy in this clinical scenario [7], and recent data from a randomized controlled trial (RCT) suggest that they effectively reduce the risk of ascites and clinical decompensation in patients with small varices [57].

Given the abovementioned data, it has been suggested that NSBBs, statins, and oral antibiotics (rifaximin [58] or norfloxacin) could be used in combination to prevent clinical decompensation in patients with cirrhosis [59]. In a recent study, patients treated with rifaximin added to propranolol showed a more marked decreased in HVPG as compared to patients on propranolol alone [60].

A further group of drugs showing promising results is represented by anticoagulants. Contrarily to what was previously thought, cirrhosis can be considered a pro-coagulant state, and experimental data suggest that low molecular weight heparin [61] and direct oral anticoagulants [62] inhibit fibrogenesis and decrease portal pressure in cirrhosis. A small RCT using enoxaparin to prevent portal vein thrombosis in patients in the waiting list for liver transplantation showed a reduction in mortality [63].

Given the increased susceptibility to life-threatening bacterial infections observed in patients with decompensated cirrhosis [8], the reduction of intestinal bacterial translocation by antibiotic therapy is another potential treatment able to reduce the risk of spontaneous bacterial peritonitis and mortality in patients with decompensated cirrhosis and ascites. In a recent RCT [64], norfloxacin combined to standard medical therapy improved survival compared with standard medical therapy alone in patients with decompensated alcoholic cirrhosis and severe liver failure. In addition, a further strategy aimed at improving effective intravascular volemia by using weekly administration of intravenous albumin in addition to standard medical therapy improved survival in patients with ascites versus standard medical therapy alone [65]. Nevertheless, these results are not yet published in full and require validation.

Transjugular intrahepatic portosystemic shunt (TIPS) is a well-accepted therapy to prevent rebleeding in patients experiencing more than one episode of variceal bleeding, in patients with refractory ascites it demonstrated a survival benefit vs. large volume paracentesis. Recent data suggest that TIPS may also be applied to other clinical scenarios in cirrhosis to improve outcomes. In a RCT of TIPS versus standard medical plus endoscopic therapy in patients presenting with variceal bleeding and poor liver function (Child–Pugh score B9 to C12 points), the early use of TIPS (within 72 hours with the aim of preventing early rebleeding) reduced mortality by 25% [66]; these results have been validated in a second multicentric study [67]. In the setting of patients with recurrent (not refractory) ascites, TIPS improved survival by over 40% in comparison to standard medical therapy [68].

A major gap remains regarding the ability to non-invasively monitor the effect of therapy on portal pressure. None of the currently available non-invasive tests holds sufficient accuracy in mirroring the HVPG response. A recent study suggested that changes in spleen stiffness (measured by point shear wave elastography) might parallel changes in HVPG and portal pressure gradient after NSBB and TIPS [69]; however, these results require validation. The development of other non-invasive tests, such as subharmonic aided pressure estimation on contrast-enhanced ultrasound [70], as well as non-invasive measurements derived by parameters from contrast-enhanced ultrasound [71] or magnetic resonance imaging [34] are urgently needed in this field.

Finally, a novel challenge has resulted with regards to the population of patients with HCV cirrhosis in whom the virus was successfully cured by direct acting antivirals. A minority of these patients will improve after treatment, but a substantial proportion (over 70%) of those who had CSPH at the time of therapy remains at risk of developing complications of portal hypertension [72]. Unfortunately, we currently lack non-invasive surrogates of HVPG in this population, and it remains unknown whether cirrhosis will successfully revert in the long term. The ‘point of no return’ in the natural history of cirrhosis is currently unknown, and certainly represents a major field for future research as well as a potential endpoint for novel therapies.

## Conclusions and future perspectives

Currently, cirrhosis is considered a dynamic disease able to progress and regress. In this new way of understanding the spectrum of changes characterizing ACLD, early diagnosis, prior to the occurrence of decompensation, is an important step to achieve a reduction in mortality due to CLD since several different pharmacological and non-pharmacological approaches can be used during this phase to prevent decompensation (an ominous step in the natural history of this disease). Ultrasound elastography of the liver allows an accurate non-invasive identification of patients with ACLD, with the additional advantage of providing a numerical surrogate of the risk of portal hypertension and complications. Prevention of decompensation is possible by reducing portal pressure through measures aimed at eliminating all the possible sources of injury (etiology and cofactors), at reducing intrahepatic resistance (e.g., by correcting intrahepatic endothelial dysfunction), and at reducing portocollateral flow. Long-standing drugs, such as NSBBs, remain the mainstay for portal pressure reduction and are able to prevent not only variceal bleeding, but also other more frequent decompensating events such as ascites. After decompensation, therapy should be aimed towards avoiding further decompensation and death, with statins being promising in these cases. TIPS is effective in decreasing the risk of variceal rebleeding and improves mortality in patients with recurrent and refractory ascites. The extent to which modulating the gut microbiota impacts the natural history of decompensated cirrhosis remains unknown, yet antibiotics already play an important role in the prevention and treatment of severe bacterial infection in decompensated patients. Unfortunately, despite the indubitable improvement in the management of portal hypertension, severe liver failure cannot be reversed.

Effective artificial liver support remains a major unmet need in patients with end-stage liver disease, with liver transplantation representing the only available curative option to date (in those who have no contraindications). Indeed, research in the field of regenerative medicine represents a major expected breakthrough of the 21st century, holding great promise [73] for a reduction in the need of liver transplantation in the future.
